# Canonical Wnt Signaling (Wnt/β-Catenin Pathway): A Potential Target for Cancer Prevention and Therapy

**DOI:** 10.29252/ibj.24.5.264

**Published:** 2020-04-29

**Authors:** S Mahmoud A Najafi

**Affiliations:** Department of Cell and Molecular Biology, School of Biology, College of Sciences, University of Tehran, P.O. Box 14155-6455, Tehran, Iran

**Keywords:** β-catenin, Human cancers, Wnt signaling

## Abstract

Precise regulation of signal transduction pathways is crucial for normal animal development and for maintaining cellular and tissue homeostasis in adults. The Wnt/Frizzled-mediated signaling includes canonical and non-canonical signal transduction pathways. Upregulation or downregulation of the canonical Wnt signaling (or the Wnt/β-Catenin signal transduction) leads to a variety of human diseases, including cancers, neurodegenerative disorders, skin and bone diseases, and heart deficiencies. Therefore, Wnt/β-Catenin signal transduction is a potential clinical target for the treatment of not only human cancers but also some other human chronic diseases. Here, some recent results including those from my laboratory highlighting the role of Wnt/β-Catenin signal transduction in human cancers will be reviewed. After a brief overview on canonical Wnt signaling and introducing some critical β-Catenin/TCF-target genes, the interaction of canonical Wnt signaling with some common human cancers will be discussed. In the end, the different segments of the aforesaid signaling pathway, which have been considered as targets for clinical purposes, will be scrutinized.

## INTRODUCTION

**Fig. 1:**
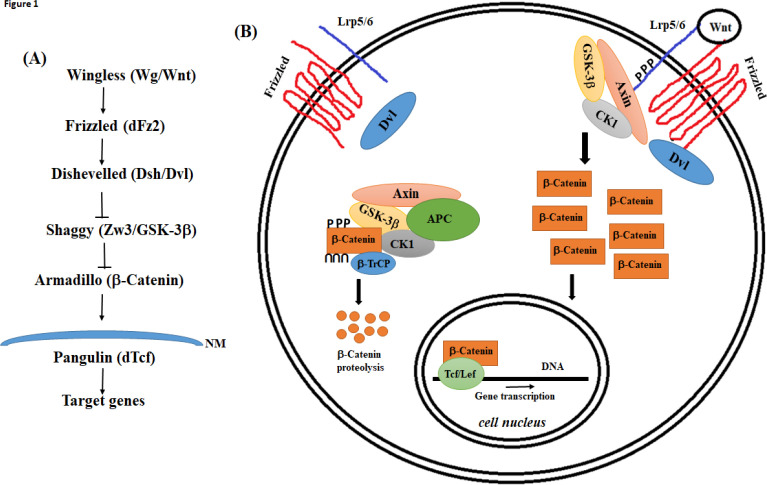
(**A**) The original model for Wg signaling in *Drosophila*, the core of the current model for the canonical Wnt signal transduction (the Wnt/β-Catenin pathway). (**B**) The simplified model for the canonical Wnt signalling in vertebrates. In the absence of the Wnt ligands, the destruction protein complex (including APC, Axin, GSK-3β, CK1, β-Trcp, and β-Catenin) works in favour of β-Catenin phosphorylation and its subsequent ubiquitin-mediated proteolysis (u letters on β-Catenin in the destruction complex). β-Catenin phosphorylation occurs at some serine and threonine residues located at amino-terminal segment of the protein (p letters on β-Catenin in the destruction complex). The protein kinases, GSK-3β and CK1, are responsible for the phosphorylation of β-Catenin in the destruction complex. In the presence of the Wnt proteins and activation of the Frizzled receptors, some components of the destruction complex, including Axin and GSK-3β, are recruited to the cell membrane via Dvl protein and form a protein complex, called “signalosome”.^[23]^ Phosphorylation of LRP co-receptor plays an important role in formation and stabilization of signalosome. Dissociation of the destruction complex leads to a significant decrease in β-Catenin phosphorylation; therefore, this protein accumulates in the cell. Increase in β-Catenin cellular levels may lead to its nuclear translocation and its interaction with the TCF/Lef transcription factors. Transcriptional regulation of some very important cellular genes appears to be the final outcome of β-Catenin nuclear translocation. NM, nuclear membrane; APC, adenomatous polyposis coli; Axin, axis inhibitor; CK1, casein kinase 1; GSK-3β, glycogen synthase kinase-3 beta; Lef, lymphoid enhancer factor; LRP5/6, low-density lipoprotein receptor-related protein 5/6; β-TrCP, beta-transducin repeat containing protein; TCF, T-cell factor

**Fig. 2:**
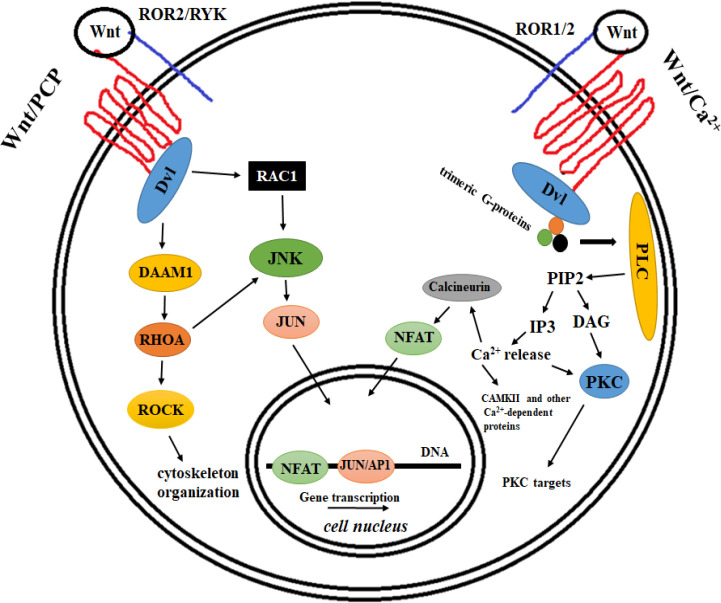
Two well-known non-canonical Wnt signalling pathways, including Wnt/Ca^2+^ (right) and Wnt/PCP (left). There are results supporting that both pathways are dependent on heterotrimeric G-protein signaling^[20,21]^. While LRP5/6 appears to work specifically for canonical Wnt signaling (Fig. 1), the co-receptors for non-canonical Wnt pathways belong to the family of RTKs (ROR1/2 and RYK). The Wnt/Ca^2+^ pathway functions through the G-proteins that activate certain isoforms of the enzyme, PLC. Activation of PLC converts the phospholipid, PIP_2_, to DAG and IP3, which are two very important cellular second messengers. DAG is a direct activator of PKC, a multifunctional protein kinase in the cell, involved in the regulation of several cellular processes. IP3, on the other hand, interacts with its receptors on endoplasmic reticulum and leads to the intracellular release of calcium. Calcium is a co-factor for the activity of many important cellular proteins, including PKC, CamKII (Ca²/calmodulin-dependent protein kinase II), and Calcineurin. As a protein phosphatase, Calcineurin can activate some transcription factors, including NFAT and therefore take part in the regulation of transcription of some important cellular genes. The other non-canonical Wnt signaling indicated in the Figure is a critical pathway involved in determining PCP. Wnt/Frizzled-mediated activation of this pathway with the help of the co-receptors (ROR2/RYK) signals through Dvl and heterotrimeric G-proteins (not shown in the Figure) to activate small GTPases like RhoA and Rac1. Activation of these small GTPases at least has two biological outcomes, reorganization of cell cytoskeleton and transcriptional regulation of some very important cellular genes mediated by transcription factors like c-Jun and AP1, a heterodimer of c-Jun and c-Fos transcription factors. DAAM1, disheveled-associated activator of morphogenesis 1; JNK, c-Jun N-terminal kinase; ROCK, Rho-associated *protein* kinase; PCP, planar cell polarity; PLC, phospholipase C; DAG, diacyl glycerol; NFAT, nuclear factor of activated T-cells; PIP2, phosphatidylinositol 4, 5-bisphosphate; IP3, inositol 1,4,5-trisphosphate

The Wnt/Frizzled-mediated signal transduction includes several signaling pathways, which have collectively been divided into two groups of biological processes based on the involvement of the β-Catenin protein^[^^1 ▶^^,^^2 ▶^^]^. The original model for Wnt signaling pathway had a central component called β-Catenin, the homolog of Armadillo protein in *Drosophila*^[^^3 ▶^^,^^4 ▶^^]^. Today, this signaling pathway has been named “the canonical Wnt signaling” or “the Wnt/β-Catenin” pathway^[^^5 ▶^^-^^7 ▶^^]^. For years, it has been thought that Wnt signaling is only one pathway (Fig. 1). Now, we have learned that the signals transmitted through the Wnts and their cognate receptors (Frizzled proteins) lead to at least three important biological processes, which two of them apparently are not directly dependent on β-Catenin protein^[^^1 ▶^^,^^2 ▶^^] ^(Fig. 2). These two signaling pathways are called “non-canonical”. This article mainly discusses the canonical Wnt signaling and its potential role in human cancers. However, it is worth mentioning that the deregulation of the non-canonical Wnt pathways also occurs in human malignancies, and interestingly, based on recent results the non-canonical Wnt signaling pathways have an important function in the survival, invasion, and metastasis of some human cancers^[^^7 ▶^^-^^9 ▶^^]^. 

The first indication of the interaction between Wnt signaling pathways and cancer was discovered in Harold Varmus’s laboratory where it was found that the retroviral integration-mediated activation of a gene called *int1* led to mammary tumor formation in mice^[^^10 ▶^^,^^11 ▶^^]^. The gene *int1* was detected to be very similar to the *Drosophila* segment polarity gene, *wingless*. Then scientists combined the names of these two genes and chose *wnt1* as the first vertebrate homolog of *wingless*. Human genome encodes 19 different Wnt proteins and 10 different Frizzled receptors^[^^12 ▶^^]^. Although it has been reported that some of these proteins work specifically for either canonical or non-canonical Wnt signaling, there are some results showing that a number of Wnt proteins (like Wnt-5a) can activate both types of Wnt pathways^[^^13 ▶^^]^. The specificity of the signals via Wnt proteins probably depends on many proteins, which regulate canonical and non-canonical Wnt signaling pathways, especially the regulators at upstream levels. Both heterotrimeric G-protein-coupled receptors and Frizzleds demonstrate a high level of structural similarities as they contain seven hydrophobic transmembrane domains^[^^14 ▶^^]^. There is sufficient evidence for the involvement of G-proteins in the regulation of both canonical and non-canonical Wnt pathways^[^^15 ▶^^-^^22 ▶^^].^ The regulatory role of heterotrimeric G-proteins can probably help us to find out more about the specificity of the signals through Wnt/Frizzled proteins. Also, this very important discovery that Frizzled proteins are not the only receptors for Wnt ligands adds another level of complexity to the Wnt-mediated signaling pathways. LRP5/6 and the receptor tyrosine kinases, ROR/RYK, are the known co-receptors for canonical and non-canonical Wnt pathways, respectively^[^^5 ▶^^,^^6 ▶^^]^.

**The current model for the canonical Wnt signaling**

According to the present model, in the absence of the Wnt ligands, a destruction protein complex, including APC, Axin, GSK-3β, CK1, and β-TrCP, maintains cellular β-Catenin protein at physiological levels^[^^5 ▶^^,^^6 ▶^^,^^23 ▶^^]^. This behavior is due to the phosphorylation of β-Catenin by CK1 and GSK-3β at serine and threonine residues located within the amino terminal of the protein (encoded by exon 3 of *CTNNB1*, the β-Catenin-encoding gene). This phosphorylation leads to 

ubiquitination and proteasome degradation of β-Catenin. Upon the interaction of the Wnt proteins to their receptors (Frizzled and LRP5/6), the destruction complex dissociates (at least partially), and a new protein complex forms at the cell membrane (called signalosome), which contains some of the components of the destruction complex^[^^23 ▶^^]^. Formation of signalosome results in a decrease in β-Catenin phosphorylation, followed by the cellular accumulation of this protein (Fig. 2). 

**A brief discussion of some Wnt/β-Catenin target genes**

Wnt/β-Catenin target gene promoters contain WREs as a part of their regulatory sequences^[^^24 ▶^^]^. WREs are the binding sites for the β-Catenin/TCF complex^[^^24 ▶^^]^. Activation of Wnt/β-Catenin signaling either increases or decreases the expression of some cellular genes. The number of genes regulated by the Wnt/β-Catenin signaling is probably cell context-dependent. In normal human cells, especially epithelial tissues, the activity of Wnt/β-Catenin signaling is extremely low (due to very low levels of cytoplasmic β-Catenin), and higher activation of this signaling pathway is limited to the situations like tissue regeneration and human malignancies^[^^25 ▶^^,^^26 ▶^^]^.

***CCND1***

This gene encodes Cyclin D1, one of the critical components of the G1-S transition of cell cycle in all animal cells^[^^27 ▶^^,^^28 ▶^^]^. Cyclin D1 has been considered as R factor^[^^27 ▶^^]^. The cellular concentration of R factor should reach a certain level before the progression of cell cycle from G1 phase to S phase. Cyclin D1 mainly interacts with the G1-S transition protein kinases, CDK4 and CDK6. Activation of these two protein kinases is required to progress cell cycle toward the S phase^[^^27 ▶^^,^^28 ▶^^]^. Deregulation of cell cycle is a feature of nearly all human cancers^[^^27 ▶^^,^^28 ▶^^]^. Cancer cells use several mechanisms to deregulate the cell cycle. Increase in *CCND1* gene expression and/or protein stabilization is among these mechanisms. The mitogenic signals via receptor tyrosine kinases together with PI3-kinase/AKT signaling are the most known pathways to enhance gene expression and protein stability of Cyclin D1^[^^29 ▶^^]^. In addition, the gene-encoding Cyclin D1 is a target of the Wnt/β-Catenin pathway. Therefore, cancer cells with upregulated Wnt/β-Catenin signaling are expected to have higher levels of Cyclin D1 protein and therefore higher cell proliferation rate.

***c-MYC***

This gene is one of the most potent cellular proto-oncogenes and encodes c-Myc protein. c-Myc is a transcription factor that binds to DNA (via helix-loop-helix and leucine zipper domains) and regulates the expression of the genes involved in many cellular functions, including cell proliferation and DNA replication^[^^30 ▶^^-^^33 ▶^^]^. c-Myc can heterodimerize with other transcription factors like Max to increase the number of the target genes. It is estimated that c-Myc is involved in the expression of more than 15% of cellular genes supporting the role of this protein in many cellular functions^[^^33 ▶^^]^. c-Myc binds to the enhancer box sequences on DNA, and by recruiting important proteins like histone acetyltransferases, it regulates gene expression^[^^32 ▶^^,^^33 ▶^^]^. c-Myc activates cell growth by upregulating the expression of ribosomal RNAs and proteins and also by decreasing the expression of the genes encoding pro-apoptotic Bcl-2 proteins^[^^30 ▶^^,^^32 ▶^^]^. c-Myc is also required for cancer cell metabolism and stemness of cancer stem cells^[^^30 ▶^^,^^32 ▶^^]^. Increase in c-Myc expression and activity has been observed in a wide range of human cancers^[^^30 ▶^^-^^33 ▶^^]^. In addition to the Wnt/β-Catenin pathway, some other mitogenic signals (like epidermal growth factor receptor and sonic hedgehog pathways) can enhance the c-Myc expression. In normal cells, the mRNA and protein levels of c-Myc are low, and this is due to the fact that c-Myc mRNA and protein are unstable^[^^30 ▶^^]^. Since the overexpression of c-Myc in normal cells can lead to oncogenic shock and induce apoptosis, cancer cells should probably obtain new features to tolerate higher levels of c-Myc activation.

***c-JUN***

The product of this gene is also a transcription factor^[^^34 ▶^^]^. c-Jun can heterodimerize with c-Fos to form a new transcription factor called AP1^[^^34 ▶^^,^^35 ▶^^]^. The expression and function of c-Jun are dependent on several signaling pathways mediated by some growth factors, pre-inflammatory signals, oxidative stress, and ultraviolet radiation^[^^35 ▶^^]^. The promoter region of *c-JUN* is responsive to the combinatorial transcription factor, AP1, which can be considered as a positive feedback for c-Jun expression^[^^35 ▶^^]^. One of the target genes of c-Jun is *CCND1*, which encodes Cyclin-D1^[^^34 ▶^^]^. Since *CCND1* is also a direct target of the Wnt/β-Catenin pathway, it can be concluded that Wnt/β-Catenin signaling has a powerful cell proliferation effect. A target gene that its expression is negatively affected by AP1 is *TP53*, the gene encoding the tumor suppressor, p53^[^^36 ▶^^]^. p53 is a known mediator of cell cycle arrest and apoptosis. Therefore, it appears that c-Jun not only induces cell proliferation but also can make cells resistant to cell cycle arrest and apoptosis by inhibiting the expression of p53. These features of c-Jun may have a significant role in tumor growth and tumor resistance to chemotherapy.

***MMP7***

This gene encodes MMP7, which belongs to the family of matrix metalloproteinases and is involved in the degradation of the extracellular matrix. In normal situations, these proteins have an important role in embryogenesis, wound healing, and tissue regeneration^[^^37 ▶^^]^. However, deregulated expression and/or function of MMPs (including MMP7) can lead to angiogenesis, cancer invasion, and metastasis^[^^37 ▶^^]^. MMP7 is normally translated as a pro-enzyme (28 kDa), which is then processed to the active form of MMP7 (19 kDa). It has been demonstrated that the overexpression of MMP7 enhances cancer invasion and metastasis^[^^37 ▶^^-^^39 ▶^^]^. Immunohistochemistry experiments have also indicated that cancer cells compared to the corresponding normal cells may express higher levels of MMP7 protein^[^^40 ▶^^]^.

***VEGF***

This gene encodes VEGF protein, which was originally identified as a hormone for growth and proliferation of endothelial cells^[^^41 ▶^^]^. Upregulation of this protein is very important for tumor growth and angiogenesis^[42]^. During animal development, VEGF stimulates embryonic vascular formation, and postnatally, this protein acts in neo-vascularization upon injuries or blockade of veins or capillaries^[^^41 ▶^^,^^42 ▶^^]^. It has also been demonstrated that VEGF enhances vascular permeability^[^^41 ▶^^]^. The function of this protein in cancer growth has drawn attention since without this protein the tumors cannot grow beyond a certain size. It has been shown that about 50% of colorectal cancer tissues express higher levels of VEGF protein compared to the corresponding normal cells^[^^43 ▶^^]^. Moreover, the expression of VEGF in colorectal tumor indicates poor prognosis and poor response to the therapy^[^^43 ▶^^]^. VEGF receptor belongs to the family of receptor tyrosine kinases, which are known as potent transducers of mitogenic signals. One of the inducers of VEGF expression and release is hypoxia^[^^42 ▶^^]^. Hypoxia increases the expression of hypoxia-inducible factor, a transcription factor whose activity can lead to VEGF release and its binding to the receptor^[^^42 ▶^^]^. The above information reveals that VEGF expression by Wnt/β-Catenin signaling can have an important role in tumor growth, tumor angiogenesis, and tumor invasion. 

***FGF18***

This gene encodes FGF18 protein, which is an important cellular mitogenic growth factor^[44]^. FGF18 is involved in animal development and tissue regeneration by regulating different cellular functions, including growth, proliferation, and survival^[^^44 ▶^^]^. When deregulated, FGF18 promotes cancer growth, angiogenesis, and metastasis^[^^45 ▶^^]^. The receptor for FGF18 is FGFR3, a member of the family of mitogenic receptor tyrosine kinases, which functions through Ras and Map kinases^[^^44 ▶^^]^. It is worth mentioning that FGF family of growth factors include at least 22 members (FGF1-FGF22), which some of them are preferentially expressed during embryonic development^[^^46 ▶^^]^. One of the known functions of FGF18 is growth and development of cartilage and bone during embryogenesis and post-embryonic development^[^^44 ▶^^,^^45 ▶^^]^. In mice, overexpression of FGF18 leads to cartilage thickness^[^^47 ▶^^]^. *FGF18* null mice die very early after birth, probably due to the deformation of the ribs^[^^47 ▶^^]^. Although FGF18 is a potent mitogen for osteoblasts and chondrocytes, this protein can also enhance the growth and proliferation of other cells, including neurons, intestine, and liver cells^[^^46 ▶^^]^. It has been reported that there is a positive association between FGF18 expression and development of some human cancers, including colon cancer^[^^45 ▶^^]^. Interestingly, it has been observed that in slow proliferating colon cancer cell lines (like Caco-2 and LT-97), addition of FGF18 to the cell culture can elevate cell proliferation^[^^45 ▶^^]^.

***c-MET***

The product of this gene, c-Met, which has also been called “HGFR” is a receptor tyrosine kinase^[^^48 ▶^^]^. c-Met is a key player in animal development, and therefore, homozygous deletion of *c-Met* gene, similar to the gene encoding the c-Met ligand (HGF), is embryonically lethal in mice^[^^48 ▶^^,^^49 ▶^^]^. HGF is mainly expressed by mesenchymal cells, while c-Met (HGFR) is normally expressed by epithelial cells, which signifies that epithelial cells expressing c-Met can respond to paracrine signals from mesenchymal cells expressing HGF^[^^49 ▶^^]^. As mentioned above, c-Met is a member of the RTK family, and therefore it can potentially be involved in several biological activities. It has been displayed that upon damage to tissues like liver, kidney, and heart, the expression of c-Met in these tissues increases, suggesting that this protein has a positive role in tissue repair^[^^48 ▶^^,^^49 ▶^^]^. In addition to the known components of the RTK signaling, some other important signaling molecules such as the intracellular tyrosine kinase, Src, the regulatory subunit of PI3-Kiase (p85 protein), Phosphlipase Cβ, Ship1, and STAT3 can be activated by c-Met^[^^48 ▶^^]^. Also, the c-Met-mediated signaling can activate gene expression that some of the target genes encode matrix metalloproteinases^[^^50 ▶^^]^. Regarding the interaction with human cancers, c-Met has been considered a potent oncogene involved in cancer cell growth, invasion, and metastasis^[^^48 ▶^^,^^49 ▶^^]^. Induction of EMT is one of the mechanisms of c-Met-mediated cancer cell invasion^[^^49 ▶^^,^^50 ▶^^]^. Genetic mutations and chromosomal rearrangements can lead to the upregulation of c-Met in human cancers^[^^48 ▶^^,^^50 ▶^^]^. The genes encoding c-Met and its ligand (HGF) are located on chromosome 7, and trisomy of this chromosome has been observed in most cases of capillary renal carcinoma^[^^51 ▶^^]^. In addition, duplication of *c-MET* gene has been reported in familial forms of renal carcinoma. Studies have also indicated that the expression of c-Met and its ligand is higher in colorectal cancer tissues than that of these proteins in the corresponding normal mucosa, and this increase is normally accompanied with cancer invasion, metastasis, and patient poor prognosis^[^^51 ▶^^,^^52 ▶^^]^. In fact, c-Met has been considered as a potential clinical target for the treatment of colorectal cancer^[^^52 ▶^^]^. 

***TERT***

*TERT* is an important Wnt/β-Catenin target gene and encodes the enzyme Telomerase, a significant marker of proliferating cells, including stem cells^[^^53 ▶^^]^. Many non-proliferative somatic cells in adults do not express *TERT* and may remain Telomerase-negative until the end of their lifespan^[^^53 ▶^^,^^54 ▶^^]^. Although Telomerase is primarily known as an enzyme adding telomere repeats to the chromosome ends during chromosome replication, further studies have shown that Telomerase is a multi-functional protein and positively regulates cell proliferation^[54,55]^. Telomerase also supports chromosomal stability in cells having short telomeres^[^^53 ▶^^,^^54 ▶^^]^. Many carcinomas do not express Telomerase at early stages, likely because these tumor cells need genomic instability to progress^[^^55 ▶^^]^. However, many of these cancers re-express Telomerase at late stages, the time when further chromosomal instability may lead to cancer cell death^[^^53 ▶^^,^^55 ▶^^]^. Telomerase has been considered as a therapeutic target for the late stages of carcinogenesis or for the tumors originating from Telomerase-positive cells like leukemia and lymphoma^[^^55 ▶^^]^.

**A conclusion on the expression of the discussed genes**

It definitely cannot be expected that β-Catenin has ability to regulate all its target genes in a specific colon cancer patient or in a cell line in which β-Catenin has been overexpressed. The correct number of genes, which are regulated by the Wnt/β-Catenin pathway, is most likely dependent on the cell context^[^^56 ▶^^]^. Activation of the canonical Wnt signaling (or the Wnt/β-Catenin pathway) occurs in more than 85% of colon cancer cases in which this signaling pathway is involved in tumor formation and progression^[^^5 ▶^^-^^8 ▶^^]^. The transcriptional activities of β-Catenin protein are a hallmark of deregulation of this signaling pathway in colon cancer and some other malignancies. We have learned that each one of the target genes (some of them mentioned above) may produce a multi-functional protein, which can greatly influence cellular activities, including growth, proliferation, survival, polarity, cytoskeleton organization, and movement. Therefore, we can easily conclude that the deregulation of the Wnt/β-Catenin signaling in cells (like colon epithelial cells) can potentially lead to the formation and progression of neoplastic cells. It is worth mentioning that the transcription of some of the target genes decreases upon the activation of the Wnt/β-Catenin signaling. A good example is *CDH1*, which encodes E-Cadherin, a true marker of epithelial cells^[^^25 ▶^^,^^26 ▶^^,^^57 ▶^^]^. Decrease in E-Cadherin expression can induce EMT, which may activate a Wnt-independent β-Catenin signaling^[^^25 ▶^^,^^26 ▶^^,^^57 ▶^^]^. Another mechanism to reduce the expression of *CDH1 *by Wnt/β-Catenin is the activation of a gene called *SNAI1*, encoding a transcription factor (Snail), which negatively regulates the transcription of *CDH1*^[^^25 ▶^^,^^58 ▶^^]^.

**β-Catenin and human cancers**

Before discussing human cancers, which are dependent on β-Catenin deregulation, it is interesting to know that different tumors may use various mechanisms to upregulate β-Catenin signaling. We know that β-Catenin cellular accumulation and its transcriptional activities are not due to only activation of the canonical Wnt pathway. The mitogenic and surviving signals through RTKs and PI3-Kinas/AKT can also upregulate β-Catenin activity via phosphorylation (at serine 9) and inactivation of GSK-3β^[^^15 ▶^^,^^59 ▶^^]^ and/or the induction of EMT. We and others have also suggested that the activation of some classes of heterotrimeric G-proteins (like Gαq signaling) can cause cellular accumulation of β-Catenin and its transcriptional activities^[^^15 ▶^^,^^16 ▶^^,^^18 ▶^^,^^21 ▶^^]^. An important question is whether the β-Catenin proteins activated by these signaling pathways functionally behave similarly, or there are different species of this protein that each tumor is dependent on one or two of them. It is also important to consider that some tumors may use two or more components of Wnt signaling pathway to perhaps amplify β-Catenin activation. An example is the colon cancer cell line, HCT-116, which carries both β-Catenin-activating gene mutations together with epigenetic silencing of the sFRP encoding gene^[^^60 ▶^^,^^61 ▶^^]^. Based on the above information, it appears that β-Catenin deregulation occurs in many human cancers^[^^5 ▶^^-^^9 ▶^^]^, and the examples below are just representatives of those tumors.

***Colon cancer***

Colon cancer is perhaps the best example of the interaction between Wnt/β-Catenin signaling and human cancers and has very well been investigated over the decades^[^^5 ▶^^-^^8 ▶^^]^. All familial adenomatous polyposis patients and more than 85% of the sporadic cases of colon cancer carry genetic mutations in the *APC* gene, resulting in the complete or partial inactivation of the APC protein^[^^62 ▶^^,^^63 ▶^^]^. During Wnt/β-Catenin signal transduction, APC protein functions as a regulator and a large scaffold protein to maintain β-Catenin protein levels at physiological concentrations. Upon the inactivation of APC in colon epithelial cells, β-Catenin accumulates in the cell. This cellular accumulation of β-Catenin may result in its nuclear translocation and transcriptional activity of this protein, which appears to be the main cause of colon cancer initiation^[^^62 ▶^^,^^63 ▶^^]^. Interestingly, the remaining cases (nearly 15%) of colon cancer, which lack *APC* mutations, carry genetic or epigenetic changes in some other components of the Wnt/β-Catenin signaling pathway (like the genes encoding Axin, β-Catenin, and sFRP)^[^^60 ▶^^-^^63 ▶^^]^. Even in hereditary non-polyposis colorectal cancer patients, which initially carry genetic mutations in the genes encoding the proteins of the mismatch DNA repair system, genetic mutations (point mutations) in the β-Catenin-encoding gene (*CTNNB1*) is very common at later stages^[^^64 ▶^^]^. β-Catenin mutations normally affect the N-terminal of the protein replacing the GSK-3β or Casein kinase phosphorylation sites^[^^64 ▶^^]^. As mentioned above, deregulation of the Wnt/β-Catenin pathway is an early event in colon cancer. Clinically, this is a very important issue because targeting the biological pathways involved in the initiation of tumorigenesis can potentially block tumor formation. 

***Melanoma***

Melanocytes developmentally originate from the migration of neural crest cells and the Wnt signaling plays an essential role in the determination of melanocyte cell fate^[^^65 ▶^^,^^66 ▶^^]^. Melanoma is a type of skin tumor that originates from the basal layer of the skin and from the pigment producing cells, melanocytes. Compared to other types of skin tumors, melanoma is a more malignant one. The interaction between this tumor and Wnt/β-Catenin signaling has been known for years^[^^65 ▶^^,^^66 ▶^^]^. It has been reported that almost all forms of benign melanoma have nuclear β-Catenin^[^^65 ▶^^,^^66 ▶^^]^. Therefore, the Wnt/β-Catenin pathway appears to support proliferation and escape from senescence of early melanoma tumor cells^[^^65 ▶^^]^. It has also been shown that the advanced melanoma cells lackβ-Catenin in the nucleus^[^^65 ▶^^,^^66 ▶^^]^. These observations are very interesting as they suggest a very complex role of Wnt/β-Catenin pathway in this type of malignancy. Probably cancer stage and tumor microenvironment are important factors when studying the interaction between Wnt/β-Catenin signaling and melanoma tumorigenesis. As mentioned before, the signals through Wnt and Frizzled proteins have generally been divided into canonical (the Wnt/β-Catenin) and non-canonical pathways. Although the role of canonical Wnt signaling was originally highlighted in human cancers (perhaps due to the intensive investigation of colorectal cancers), more recent results indicate that the non-canonical Wnt pathways are also involved in human carcinogenesis^[^^5 ▶^^-^^7 ▶^^]^. Based on the current knowledge about human carcinomas (the tumors originating from epithelial tissues), in the case of the tumors dependent on Wnt signaling, it appears that the canonical Wnt pathway supports early tumorigenesis, while the non-canonical Wnt pathways help tumor invasion and metastasis^[^^5 ▶^^-^^7 ▶^^]^. In melanoma, the genetic mutations of the components of the canonical Wnt pathway (including β-Catenin itself) are rare, but the non-canonical Wnt pathway (mediated by Wnt-5a) helps tumor metastasis^[^^65 ▶^^]^. The increased intracellular levels of β-Catenin during early stages of melanoma is likely due to the activation of other signaling pathways^[^^65 ▶^^]^. The experiments performed in mice have suggested that β-Catenin-mediated melanoma formation is dependent on the activation of the proto-oncogene, N-Ras^[^^66 ▶^^]^. It has been speculated that the downregulation of β-Catenin signaling, which occurs during melanoma aggression, is possibly due to the activation of non-canonical Wnt pathways^[^^65 ▶^^-^^67 ▶^^]^. One proposed mechanism is that interaction of Wnt-5a with Frizzled 2/5 and the coreceptor, ROR2 (a receptor tyrosine kinase) activates *phosphatidylinositol* signaling, which leads to the release of calcium from intracellular stores and activation of protein kinase C^[^^13 ▶^^,^^65 ▶^^]^. The outcome of the released calcium could include the activation of calpain-mediated proteolysis of filamin, upregulation of the transcription factor, Snail, and upregulation of the cytoskeleton protein, Vimentin, which collectively enhance cell motility and transition of tumor cells to a mesenchymal phenotype^[^^67 ▶^^,^^68 ▶^^]^. It has also been revealed that Wnt-5a activation leads to the proteolysis of β-Catenin via a mechanism that is independent of GSK-3β and is through the activation of the ubiquitin ligase, SIAH2^[^^65 ▶^^]^. These results all show that the downregulation of β-Catenin occurs during melanoma tumor invasion.

***Hepatocellular carcinoma***

HCC is one of the leading cause of death from cancer in many populations, and studies have exhibited that the aberrant regulation of Wnt signaling (both canonical and non-canonical) is involved in hepatocellular carcinogenesis^[^^69 ▶^^-^^73 ▶^^]^. HCC (like many other carcinomas) is a multi-stage and complex disease that requires the accumulation of several genetic and epigenetic changes to develop. Deregulation of the canonical Wnt signaling (and therefore the upregulation of β-Catenin) in HCC is very common and occurs in nearly 95% of the cases^[^^70 ▶^^,^^71 ▶^^]^. It appears that a relatively similar mechanism to what discussed for melanoma applies for HCC as well, that the canonical Wnt signaling (the Wnt/β-Catenin pathway) is involved in the initiation of hepatocellular tumorigenesis, while the non-canonical Wnt pathway helps tumor advancement and invasion^[^^71 ▶^^]^. Therefore, a combination of Wnt signaling pathways can support the proliferation, survival, migration, and invasiveness of hepatocytes^[^^69 ▶^^-^^72 ▶^^]^. The molecular events leading to the activation of β-Catenin in HCC include *CTNNB1* and *AXIN* gene mutations, *WNT3*/*FZD7* overexpression, and *sFRP1/5* repression^[71]^. *APC* gene mutations in HCC are rare and include only 1% to 3% of cases^[^^71 ▶^^]^. It has been estimated that between 40% to 70% of HCC cases have nuclear β-Catenin accumulation. It is interesting to mention that β-Catenin gene (*CTNNB1*) mutations normally occur late in HCC tumorigenesis, but the cellular accumulation of β-Catenin is an early event^[^^71 ▶^^]^. This means that the early cellular accumulation of β-Catenin in HCC is probably not due to the mutations in the β-Catenin-encoding gene. The above information also suggests that the late β-Catenin mutations probably have an extra role in HCC tumorigenesis. A balanced β-Catenin signaling is important for liver tissue homeostasis, and therefore the deregulation of Wnt/β-Catenin pathway probably has an important role in the initiation of HCC^[^^69 ▶^^-^^72 ▶^^]^. However, studies in mice have shown that β-Catenin upregulation *per se* is not sufficient to initiate hepatoma, and other pathways like H-Ras signaling are also required for the initiation of the tumor^[^^71 ▶^^]^. Also, by using conditional knockout experiments, it has been reported that diethyl-nitrosamine-induced HCC in mice can be enhanced severalfold either in the absence of wild type β-Catenin or in the presence of a mutant β-Catenin^[^^69 ▶^^,^^71 ▶^^]^. These results have made it difficult to conclude that Wnt/β-Catenin signaling is involved in the initiation of HCC tumorigenesis, but more recent results have indicated that both types of Wnt signaling pathway (canonical and non-canonical) help HCC aggressiveness and resistance to therapy and also provide an appropriate microenvironment to support tumor growth and survival^[^^69 ▶^^,^^70 ▶^^,^^73 ▶^^]^.

***Pancreatic cancer***

PDAC is among the deadliest human cancers with an overall survival rate of about 8%^[^^74 ▶^^-^^77 ▶^^]^. The metastasis is relatively common and more than half of the patients have already distant metastases at the time of diagnosis^[74]^. Activating mutations in the *KRAS* gene have been detected in more than 95% of the cases^[^^74 ▶^^,^^75 ▶^^]^. Also, inactivation of the tumor suppressors like p53, INK4a, and DPC4 is documented in pancreatic cancer progression and development^[^^74 ▶^^]^. In addition, deregulation of signaling pathways like those mediated by Hedgehog, Notch, and Wnt may occur in pancreatic cancer, which has an important role in the progression of the tumor^[^^75 ▶^^]^. Nuclear accumulation of β-Catenin has been detected in moderate to poorly differentiated PDAC, and it has been noted that nuclear β-Catenin accompanies with poor prognosis^[^^74 ▶^^]^. Meanwhile, the genetic mutations of downstream components of the canonical Wnt pathway are not very common in pancreatic cancer^[^^74 ▶^^,^^76 ▶^^,^^77 ▶^^]^. This means that probably molecular changes affecting the upstream levels of Wnt signaling are acting in β-Catenin upregulation. In fact, overexpression of Wnts and Frizzled receptors has been reported in PDAC^[^^75 ▶^^]^. It has been shown that the upregulation of β-Catenin during mice development leads to pancreatoblastoma, while upregulation of this protein after birth results in PDAC^[^^74 ▶^^,^^75 ▶^^]^. Interestingly, several β-Catenin target genes (including *Gli2*, *Id2*, *Vegfc*, and *Cyr61*) have been linked to the tumorigenesis of PDAC. In this sense, the role of CYR61 has been highlighted in malignant potential of PDAC^[^^74 ▶^^]^. Higher expression of CYR61 has been detected in about 85% of PDAC cases, and expression of this gene has been associated with the later stages of the disease^[^^74 ▶^^]^. It has been suggested that CYR61 binds to LRP6 and activates Wnt/β-Catenin pathway using a positive feedback loop^[^^74 ▶^^]^. Although the role of non-canonical Wnt pathways in PDAC development is unclear, studies have revealed that Wnt-5a-mediated signaling is involved in the transformation of pancreatic cancer cells^[^^74 ▶^^]^.

**Clinical targeting of the Wnt/β-Catenin signaling**

Due to the interaction between Wnt/β-Catenin signaling and human carcinogenesis, this signaling pathway has been considered as a potential clinical target for the prevention and treatment of a large number of human cancers, especially those dependent on Wnt/β-Catenin signaling at early stages (like colon cancer). However, targeting such an important signaling pathway without clinical side effects seems to be difficult. This issue gets more serious when there are cross-talks between Wnt/β-Catenin signaling and other signaling pathways. After about 40 years of intensive research on Wnt signaling^[^^78 ▶^^]^, there are still some unanswered questions about the regulation of this signaling pathway. Despite these limitations, several compounds have been introduced as candidates to downregulate Wnt/β-Catenin signaling in human cancers. Although none of these compounds has yet been approved for clinical use, some have produced promising results in pre-clinical studies. Some of these compounds and their specific targets have been briefly discussed below.

**β-Catenin/TCF**

Perhaps the most specific segment of Wnt/β-Catenin signaling is the interaction of β-Catenin with TCF/Lef family members and the transcriptional activities of this protein complex. Although the genes regulated by the complex of β-Catenin/TCF may vary based on the cell-context, specific targeting of this protein complex appears to be promising. Currently, several compounds, including PKF115-584, CGP049090, iCRT3, iCRT5, iCRT14, PNU-74654, and BC21, against β-Catenin/TCF complex are under preclinical investigations^[^^79 ▶^^-^^82 ▶^^]^.

**Tankyrase 1/2**

Several compounds, like XAV939, IWR, and G007-LK, have been designed to upregulate Axin, an inherent component of the Wnt/β-Catenin signaling pathway^[^^6 ▶^^,^^81 ▶^^,^^82 ▶^^]^. These compounds are, in fact, the inhibitors of the enzyme, Tankyrase. Tankyrase 1/2 is involved in ADP-ribosylation of Axin and therefore destabilizes this protein via inducing its ubiquitylation and degradation^[^^6 ▶^^,^^81 ▶^^,^^82 ▶^^]^. As mentioned above, Axin, as a second scaffold protein (after APC), functions as a negative regulator of β-Catenin stability.

**Porcupine**

Appropriate synthesis and secretion of Wnt proteins have crucial roles in the activation of both canonical and non-canonical Wnt signaling pathways^[^^6 ▶^^]^. Secretion of Wnt ligands is a very complex process, and for most Wnt proteins, glycosylation and lipid modification (acylation) are prerequisites for appropriate vesicular trafficking and secretion^[^^5 ▶^^,^^6 ▶^^]^. Porcupine is a membrane protein with O-acyltransferase activity and is involved in palmitoylation (at a serine residue) and maturation of most Wnt proteins^[^^6 ▶^^,^^83 ▶^^]^. It has been demonstrated that the palmitoylated serine is one of the interacting sites of the Wnt proteins to the extracellular cysteine-rich domain of Frizzled receptors^[^^6 ▶^^,^^83 ▶^^]^. It has also been shown that in the absence of Porcupine, the Wnt proteins cannot be secreted and are trapped in endoplasmic reticulum^[^^83 ▶^^]^. Based on these data, it can be concluded that Porcupine is a potential clinical target although it seems likely that the blockade of Porcupine affects the secretion of many Wnt proteins and produces non-specific and unwanted results. The compounds IWP2, LGK974, and C59 are among the blockers of Porcupine^[^^6 ▶^^,^^82 ▶^^]^.

**Other targets**

It is worth mentioning that the above targets are not the only ones that have been considered for clinical investigations to modulate Wnt/β-Catenin signaling. The other targets include DVL, Wnts, and Frizzled proteins^[^^82 ▶^^]^. The compounds like FJ9, NSC668036, and 3289-8625 have been used for preclinical studies against DVL (the human homologue for *Drosophila* Dishevelled proteins)^ [^^82 ▶^^]^. Based on the knowledge about the role of DVL proteins in regulating Wnt signaling, it is quite predictable that blocking these proteins may have a huge effect on the biological activities of many cells (including cancer cells). DVL is a critical protein involved in regulating both canonical and non-canonical Wnt signaling. DVL is, in fact, an upstream component of the Wnt pathways and has a very important role in specifying the signals through Wnt and Frizzled proteins^[^^5 ▶^^-^^7 ▶^^]^. These features of DVL proteins make them challenging targets as far as the cytotoxicity and drug side effects are concerned. A monoclonal antibody (OMP-18R5) has been generated that recognizes several Frizzled receptors and is currently in phase I clinical trial^[^^84 ▶^^]^. Application of specific monoclonal antibodies against oncogenic tyrosine kinase receptors has been proven to be successful, and some of such antibodies (like Herceptin against Her2/Neu) have been approved for clinical use. RTKs are more diverse than Frizzled proteins (58 vs. 10), and they have potential mitogenic activities. Also, overexpression of RTKs has been observed in several human cancers^[^^85 ▶^^]^, while the expression levels of Frizzled proteins in human cancers have not been extensively studied. Since there are only 10 Frizzled proteins encoded by human genome and the involvement of these proteins in both canonical and non-canonical Wnt pathways, it can be concluded that each Frizzled protein may regulate several biological activities. Therefore, for clinical purposes, targeting a Frizzled receptor with monoclonal antibodies appears to be more challenging than that for receptor tyrosine kinases. 

**Conclusions and future** **directions**

There is no doubt that the canonical Wnt signaling (or the Wnt/β-Catenin pathway) is a potential clinical target for cancer therapy. However, an important question is which segment of this pathway should be targeted to produce better and more specific results. The final step of a signaling pathway appears to be the best choice, which for the canonical Wnt pathway includes the β-Catenin-mediated gene transcription. However, the transcriptional activities of β-Catenin should be cellular context-dependent, and the β-Catenin/TCF complex may not be the only factor in specifying the target genes. Definitely, further investigations on molecular details of β-Catenin transcriptional complexes are needed to address the above question. 
